# Network Inference Algorithms Elucidate Nrf2 Regulation of Mouse Lung Oxidative Stress

**DOI:** 10.1371/journal.pcbi.1000166

**Published:** 2008-08-29

**Authors:** Ronald C. Taylor, George Acquaah-Mensah, Mudita Singhal, Deepti Malhotra, Shyam Biswal

**Affiliations:** 1 Computational Biology and Bioinformatics Group, Pacific Northwest National Laboratory, U.S. Department of Energy, Richland, Washington, United States of America; 2 Department of Pharmaceutical Sciences, Massachusetts College of Pharmacy and Health Sciences, Worcester, Massachusetts, United States of America; 3 Department of Environmental Health Sciences, Bloomberg School of Public Health, Johns Hopkins University, Baltimore, Maryland, United States of America; National Cancer Institute, United States of America and Tel Aviv University, Israel

## Abstract

A variety of cardiovascular, neurological, and neoplastic conditions have been associated with oxidative stress, i.e., conditions under which levels of reactive oxygen species (ROS) are elevated over significant periods. Nuclear factor erythroid 2-related factor (Nrf2) regulates the transcription of several gene products involved in the protective response to oxidative stress. The transcriptional regulatory and signaling relationships linking gene products involved in the response to oxidative stress are, currently, only partially resolved. Microarray data constitute RNA abundance measures representing gene expression patterns. In some cases, these patterns can identify the molecular interactions of gene products. They can be, in effect, proxies for protein–protein and protein–DNA interactions. Traditional techniques used for clustering coregulated genes on high-throughput gene arrays are rarely capable of distinguishing between direct transcriptional regulatory interactions and indirect ones. In this study, newly developed information-theoretic algorithms that employ the concept of *mutual information* were used: the Algorithm for the Reconstruction of Accurate Cellular Networks (ARACNE), and Context Likelihood of Relatedness (CLR). These algorithms captured dependencies in the gene expression profiles of the mouse lung, allowing the regulatory effect of Nrf2 in response to oxidative stress to be determined more precisely. In addition, a characterization of promoter sequences of Nrf2 regulatory targets was conducted using a Support Vector Machine classification algorithm to corroborate ARACNE and CLR predictions. Inferred networks were analyzed, compared, and integrated using the Collective Analysis of Biological Interaction Networks (CABIN) plug-in of Cytoscape. Using the two network inference algorithms and one machine learning algorithm, a number of both previously known and novel targets of Nrf2 transcriptional activation were identified. Genes predicted as novel Nrf2 targets include Atf1, Srxn1, Prnp, Sod2, Als2, Nfkbib, and Ppp1r15b. Furthermore, microarray and quantitative RT-PCR experiments following cigarette-smoke-induced oxidative stress in Nrf2^+/+^ and Nrf2^−/−^ mouse lung affirmed many of the predictions made. Several new potential feed-forward regulatory loops involving Nrf2, Nqo1, Srxn1, Prdx1, Als2, Atf1, Sod1, and Park7 were predicted. This work shows the promise of network inference algorithms operating on high-throughput gene expression data in identifying transcriptional regulatory and other signaling relationships implicated in mammalian disease.

## Author Summary

A variety of conditions including certain cancers and heart diseases, diabetes mellitus, and rheumatoid arthritis have been associated with the generation of high levels of highly reactive molecular species under conditions known as “oxidative stress.” A number of protein molecules have been identified as participants in an elaborate response to oxidative stress. Sustained elevated generation of reactive species can overwhelm this response and lead to disease conditions. In these studies, we make use of data generated from over 250 studies (microarrays) in which messenger RNA levels of the gene precursors of mouse lung proteins have been examined collectively. We have made use of computational approaches to help identify the key regulatory relationships among the proteins that respond to oxidative stress. Nrf2, a protein known as a master regulator of oxidative stress response, was a principal focus of our studies. Among the novel regulatory targets of Nrf2 we identified is Als2, a protein involved in amyotrophic lateral sclerosis (Lou Gehrig's disease). We also identify important candidate three-party regulatory relationships, one of which involves the recently discovered Srxn1, an antioxidant protein that reverses S-glutathionylation, a common posttranslational modification associated with diseases such as Parkinson's disease, diabetes, hyperlipidemia, Friedreich's ataxia, renal cell carcinoma, and HIV/AIDS. These studies demonstrate the utility of network inference algorithms and affirm that Nrf2 has a direct regulatory role over the expression of other genes responding to oxidative stress.

## Introduction

Sustained elevated levels of reactive oxygen species (ROS) have been associated with the etiology of a vast range of pathological conditions. These include a variety of neurodegenerative diseases, cardiovascular diseases, cancer, diabetes mellitus, rheumatoid arthritis, and obstructive sleep apnea [1]. ROSs are highly reactive molecules. They include the superoxide anion, the hydroxyl radical, and hydrogen peroxide. ROSs are a natural by-product of oxygen metabolism. However, ROS levels can dramatically increase during times of environmental stress, causing injury and damage by attacking DNA, protein and lipid, thereby leading to oxidative stress. A number of redox-regulated gene products serve to protect cells from such ROS damage. The antioxidant response element (ARE), a cis-acting DNA element, is known to be activated by oxidative stress and to be responsible for the transcriptional regulation of several redox-regulated gene products [2].

The principal transcription factor that binds to the ARE is Nuclear factor erythroid 2-related factor (Nrf2) [3]. Nrf2 is a basic leucine zipper (bZIP) transcription factor that translocates to the nucleus following liberation under oxidative stress conditions from its cytosolic inhibitor Keap1 [4]. In the nucleus, Nrf2 forms dimers with the proteins Maf, Jun, Fos, ATF4 and/or CBP, and regulates transcription by binding to the ARE upstream of a number of target genes [4]–[7]. Established Nrf2-regulated genes include Cu/Zn superoxide dismutase, catalase, thioredoxin, thioredoxin reductase, glutathione reductase, glutathione peroxidase and ferritin (L) [3]. All of these genes are involved in the response to oxidative stress. There are several other genes also known to be involved in the response to oxidative stress [1]. The transcriptional regulatory relationships at the mRNA level, and the signaling relationships at the protein level linking these genes and their products are only partially resolved.

To find direct regulatory targets of Nrf2, we use two algorithms that can infer such regulatory links from gene expression data: Context Likelihood of Relatedness (CLR) [8] and the Algorithm for the Reconstruction of Accurate Cellular Networks (ARACNE) [9]–[11]. These algorithms were applied to the analysis of the mouse lung gene expression datasets to infer regulatory connections between oxidative stress genes. Both of these algorithms use the concept of mutual information (MI) from information theory [12]. The pair-wise MI scores calculated are derived from correlations in the patterns of expression of the two genes involved. We also annotate and perform further analysis of the putative target set thus identified.

Data derived from the promoter regions of known Nrf2 targets were used to train LibSVM, a machine learning support vector machine classification algorithm [13]. LibSVM was then used to confirm the predictions derived from gene expression data via a separate analysis of upstream DNA sequences of the predicted target genes. We also identify signaling partners of a key Nrf2 target, NAD(P)H:quinine oxidoreductase 1 (Nqo1), shedding light on previously unidentified interactions, many of which are supported by independent microarray and quantitative RT-PCR experiments.. These results demonstrate the promise of network inference algorithms in identifying transcriptional regulatory and other signaling relationships implicated in mammalian disease.

## Results

**Figure 1:**
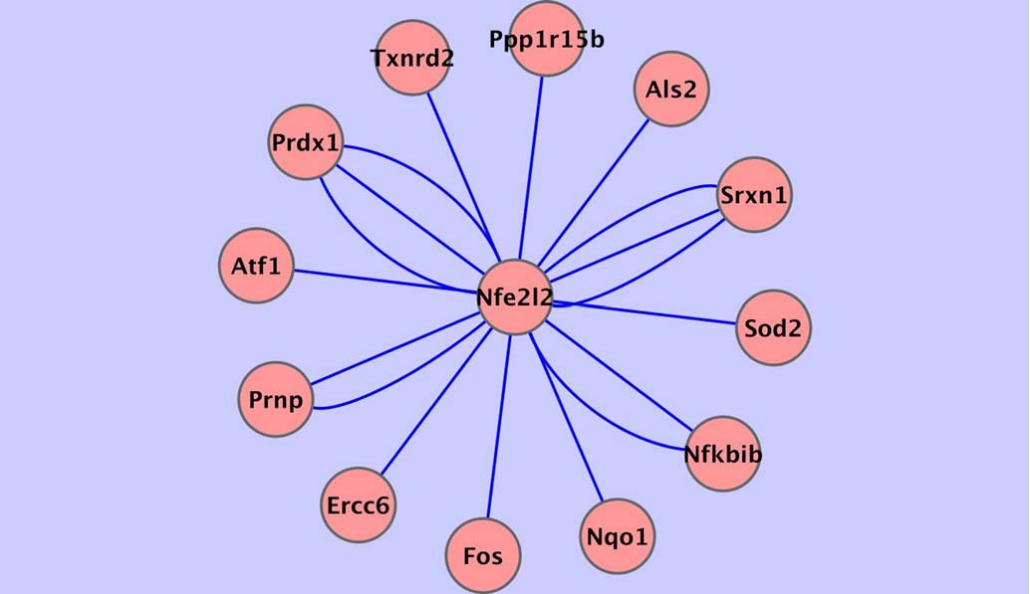
CLR algorithm results showing connections to gene Nfe212 (protein Nrf2). Regulatory interactions involving Nrf2 as determined using the CLR algorithm. Across 260 microarrays, profiles of genes categorized by the Gene Ontology as participating in the *response to oxidative stress* were examined. Z-scores were calculated on the basis of the CLR mutual information based values. At a z-score cutoff of 2.0 (two standard deviations above the mean score of all pair-wise CLR calculations), eighteen edges were reported that involved any of the Affymetrix probe sets representing the Nfe2l2 gene. These edges are shown in Figure 1. Thirteen of the eighteen putative edges had z-scores of 2.45 or higher. Some of these edges had the same gene at the other end (duplicate edges from the different Nfe2l2 probe sets), resulting in a total of twelve genes shown connected to Nfe2l2 in Figure 1 and twelve entries reported for CLR in Table 1. The nodes represent genes and the lines (edges) between them represent transcriptional regulatory relationships. Interactions involving Nrf2 (Gene Symbol: Nfe2l2) are depicted in this diagram. Multiple edges between two nodes indicate multiple array probe-sets on the arrays referencing the same gene.

Use of the two network inference algorithms, ARACNE and CLR, on the gene expression data, as well as use of the LibSVM algorithm on sequence data, yielded a number of outcomes where the same regulatory edge was predicted by all three algorithms (Table 1). ARACNE and CLR use the MI metric on the expression data to identify direct dependencies. LibSVM, trained with sequence data from upstream regions of known Nrf2-regulated genes (positive examples) and empirically determined Nrf2-independent genes (negative examples), was used to predict transcription targets from the test set of putative Nrf2-regulated target genes previously identified by ARACNE and CLR. Figure 1 depicts findings of the CLR algorithm when applied to mouse lung microarray data, with a focus on interactions involving Nrf2 determined by using (see Methods) the Collective Analysis of Biological Interaction Networks (CABIN) software [14]. A *z*-score cutoff of 2.0 on the CLR score set yielded eighteen edges above the cutoff between the probe sets representing the Nfe2l2 gene that produces Nrf2 and the probe sets for other genes in the combined dataset. In other words, the set of gene states for Nfe212 contained enough information on the states of 18 other genes (probe sets) to lift their pairwise score two standard deviations or higher above the average CLR score among all genes in the set. Given that Nfe2l2 and other genes are represented by more than one probe set, these eighteen edges yield connections from Nfe2l2 to twelve other genes.

**Table 1:** Summary of Nrf2-regulated gene target predictions.

Gene	ARACNE	CLR	LibSVM	Q RT-PCR
Als2	+	+	+	+
Atf1	+	+	+	
Crebbp	+	−	−	
Epas1	+	−	−	+
Ercc6	+	+	−	+
Fos	+	+	−	+
Hif1a	+	−	−	
Idh1	+	−	+	
Jun	+	−	−	+
Nfe2l2	N/A	N/A	+	
Nfkbib	+	+	+	
Nqo1	+	+	+	+
Park7	+	−	−	+
Ppp1r15b	+	+	+	+
Prdx1	+	+	+	
Prdx2	+	−	−	
Prnp	+	+	+	
Sod1	+	−	−	
Sod2	+	+	+	+
Srxn1	+	+	+	+
Txnrd2	+	+	−	+

+Nrf2 regulated.

−Nrf2 regulation NOT predicted.

Q RT-PCR confirmed by quantitative RT-PCR data.

**Figure 2:**
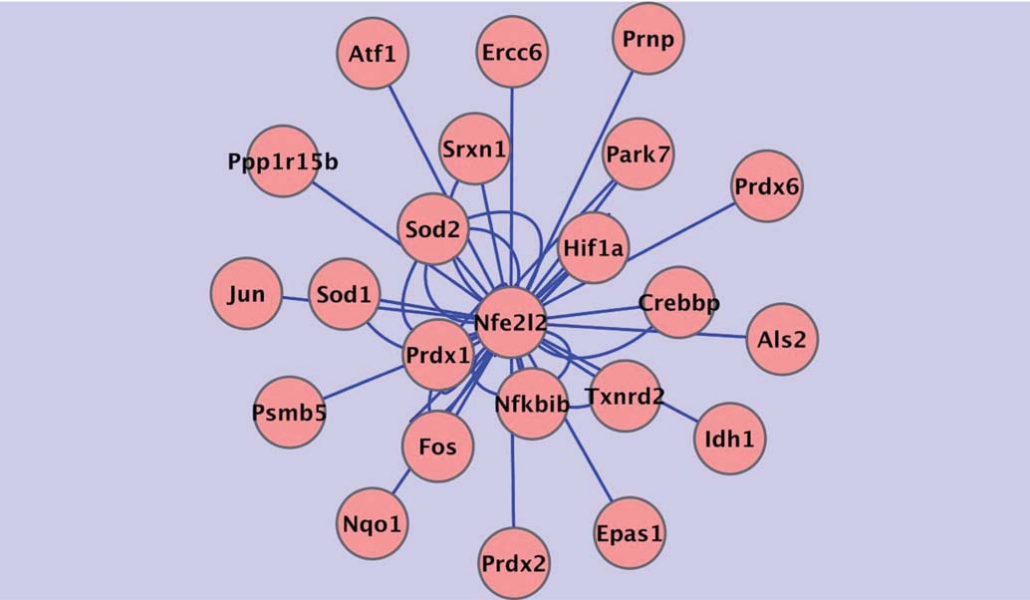
ARACNE algorithm results showing connections to gene Nfe212 (Nrf2). Regulatory interactions involving Nrf2 as determined using the ARACNE algorithm. Across 260 microarrays, profiles of genes categorized by the Gene Ontology as participating in the *response to oxidative stress* were examined. The DPI tolerance was set at 0.15; *p* = 1e-7. The nodes represent genes and the lines (edges) between them represent transcription regulation relationships. Interactions involving Nrf2 (Gene Symbol: Nfe2l2) are depicted in this diagram. Multiple edges between two nodes indicate multiple array probe-sets on the arrays referencing the same gene.

Figure 2 is a depiction of the dependencies obtained using the sets of microarrays and the ARACNE algorithm. A high significance threshold for MI values was used, with a *p*-value of 1e-7. Post-processing of the inferred edges to remove indirect regulatory relationships was done using a DPI tolerance of 0.15. For a more focused view, interactions involving Nrf2 were selected. Cutoffs for both the ARANCE and CLR algorithms were empirically determined. The cutoffs were pushed as high as possible to exclude false regulatory connections while still retrieving at least a moderate size set of interactions to explore and validate with quantitative RT-PCR, LibSVM, and literature search. In this sense, our work is classic exploratory analysis. All the Nrf2 target genes found using the CLR algorithm were also selected under the ARACNE algorithm under the cutoff values as stated above, and with the parameter settings as given in Methods. However, ARACNE also found additional putative Nrf2 targets. This finding is, however, not an indication that the dependencies identified only by ARACNE are untrustworthy. As summarized in Table 2, all of the direct dependencies predicted only by ARACNE are backed by the force of biochemical evidence. These observations underscore the power of these inference algorithms (given large enough datasets) as potential guides in the search for regulatory and signaling connections in biological networks.

**Table 2:** Previous biochemical links of several identified genes to Nrf2 or ARE.

ARACNE and LIBSVM	ARACNE and CLR only	ARACNE only	Previous evidence
Idh1			No previous evidence
	Ercc6		No previous evidence
	Fos		Jaiswal, 2004
	Txnrd2		Evidence for Txnrd1
		Crebbp	Katoh et al., 2001
		Epas1	Scortegagna et al., 2003
		Hif1a	Gong et al., 2001
		Park7	Clements et al., 2006
		Sod1	Park and Rho, 2002^a^

a Nrf2 transcription regulation target.

Seeking further evidence at the sequence level for direct, DNA-binding regulation between Nrf2 and the potential sets of target genes produced by the ARACNE and CLR runs, we used LibSVM, our selected algorithm for supervised machine learning. The training set in the classification of target gene, non-target gene consisted of features of upstream DNA promoter regions of known Nrf2 transcriptional regulation targets and empirically-determined non-target Nrf2-independent genes (Text S1). Using the LibSVM nu-SVC classifier at cost = 1, *ν* = 0.36 and *γ* = 2^−13^, a true positive rate of 0.7 or better was obtained under two cross-validation conditions for the genes in the training set. Furthermore, the precision, recall, and area under the ROC curves were 0.7 or better (Table 3). The LibSVM predictions generated on a test set obtained from the dependencies identified by CLR and ARACNE (Figures 1 and 2) posit that Atf1, Nqo1, Nfkbib, Prdx1, Srxn1, Prnp, Sod2, Ppp1r15b, Als2, Idh1, and Nrf2 (Nfe2l2) are transcriptionally Nrf2-regulated. Of these, Nqo1, Prdx1, and Nrf2 are established targets of Nrf2 transcriptional regulation [3]. Tables 1, 2, and 4 summarize our results. Table 1 shows all gene targets of possible direct Nrf2 regulation reported by either ARACNE, CLR, or LibSVM, for a total of 21 genes. Table 2 presents what was previously known about these putative target genes, based on our literature search.

**Table 3:** LibSVM performance.

	Correctly classified	TP rate^a^	FP rate^a^	Precision^a^	Recall^a^	F-measure^a^	ROC area^a^
10-fold	69.39%	0.7	0.3	0.7	0.7	0.69	0.7
Leave-one-out	71.43%	0.72	0.28	0.72	0.72	0.71	0.72
Train set only	79.59%	0.79	0.21	0.8	0.79	0.79	0.79

This scheme was used for Nrf2 regulation predictions.

Leave-one-out = *N*-fold cross-validation; 10-fold = 10-fold cross-validation.

LibSVM Details: Used nu-SVC, *ν* = 0.36, *γ* = 2^−13^, *C* = 1; train set size = 49.

TP = true positive; FP = false positive.

a Represents mean value for “Nrf2-regulated” and “Not-Nrf2-regulated” classes.

**Table 4:** Potential novel transcription regulatory targets of Nrf2 in mouse lung^a^.

Official symbol	Name
Als2	Amyotrophic lateral sclerosis 2 (juvenile) homolog (human)
Atf1	Activating transcription factor 1
Nfkbib	Nuclear factor of kappa light chain gene enhancer in B-cells inhibitor, beta
Ppp1r15b	Protein phosphatase 1, regulatory (inhibitor) subunit 15b
Prnp	Prion protein
Sod2	Superoxide dismutase 2, mitochondrial
Srxn1	Sulfiredoxin 1 homolog (*S. cerevisiae*)

a Prediction based on concurring ARACNE, CLR, and LibSVM characterization of data. Additionally, these gene products did not show up directly interacting with Nrf2 on the networks generated by our automated literature searches.

**Figure 3:**
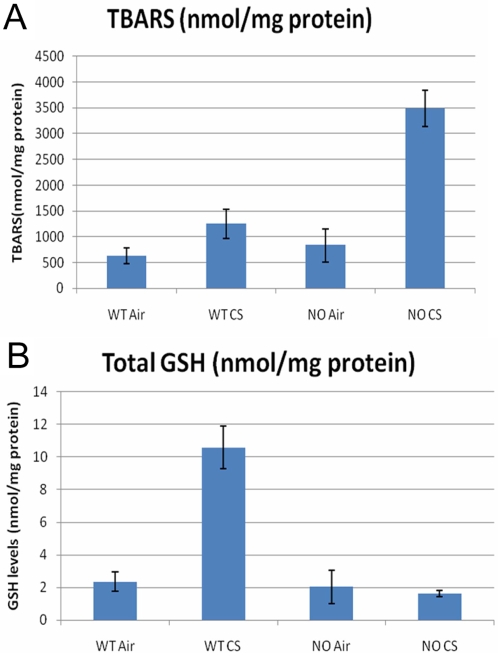
Oxidative stress markers in Nrf2^+/+^ and Nrf2^−/−^ cigarette smoke (CS)-exposed or air-exposed lungs. Figure 3A - lower induction values of total GSH were observed after CS exposure in Nrf2^−/−^ (NO) lungs after CS as compared to Nrf2^+/+^ (WT) CS exposed lungs. Figure 3B - levels of TBARS (marker of lipid peroxidation) were elevated in NOCS lungs as compared to WTCS lungs. The data is shown as Mean±SD based on three replicates (*n* = 3) in each of the four conditions.

Array experiments involving wild type (WT) and Nrf2 knockout (NO) mouse lungs were then conducted to verify the regulatory role of Nrf2 on the expression of the genes identified. The mice were exposed to either air or cigarette smoke (CS). CS-induced elevations of glutathione (GSH) and Thiobarbiturate reactive substances (TBARS) levels depicted in Figure 3 illustrate the capacity of CS to induce oxidative stress. GSH levels rise in response to oxidative stress, as a protective measure [1]. In the absence of Nrf2, the CS-induced rise in GSH levels is abolished (Figure 3A). This suggests a requirement for Nrf2 for the rise in GSH levels, and underscores the protective role of Nrf2. Increases in TBARS indicate increased decomposition of lipid peroxidation products and signal the presence of oxidative stress [15]. However in the absence of Nrf2, the CS-induced rise in lipid peroxidation as indexed by elevated TBARS levels is enhanced (Figure 3B). This emphasizes a protective role of Nrf2 against CS-induced lipid peroxidation.

**Figure 4:**
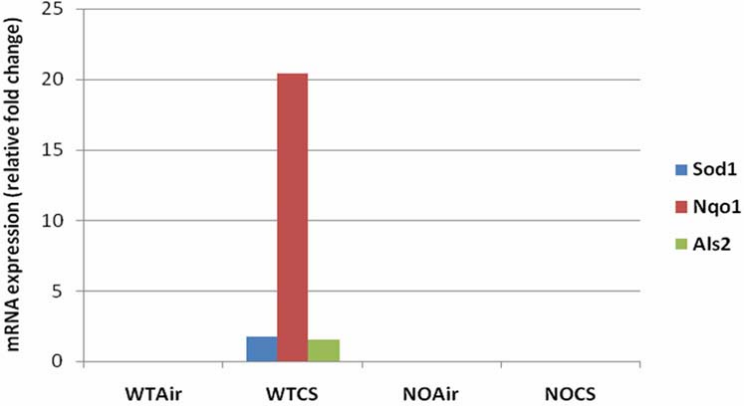
Oxidative stress-mediated induction in Sod1, Nqo1 and Als2 mRNA. Increases in expression of Sod, Nqo1, and Als2 mRNA only in Nrf2^+/+^ (WT) CD-1 mice but not Nrf2^−/−^ (NO) mice following cigarette smoke (CS) exposure. This figure depicts mean (*n* = 3) mRNA expression from the microarrays on Nrf2^+/+^ air-exposed (WTAir), Nrf2^+/+^ CS-exposed (WTCS), Nrf2^−/−^ air-exposed (NOAir) and Nrf2^−/−^ CS-exposed (NOCS). Results shown suggest regulation of these genes by Nrf2.

**Figure 5:**
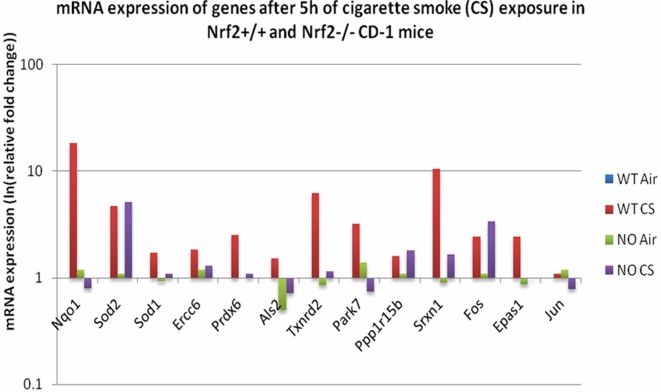
Oxidative stress-mediated induction of numerous predicted Nrf2 associated genes. Nqo1, Sod1, Ercc6, Prdx6, Als2, Txnrd2, Park7, Srxn1 and Epas1 mRNA were induced selectively more in Nrf2^+/+^ mouse lungs after CS exposure. Some of the Nrf2-associated predicted genes as Sod2, Ppp1r15b, Fos and Jun either show no differential induction or an inverse relation with Nrf2 gene. Nrf2 apparently exerts a negative regulatory influence on the expression of Sod2, Ppp1r15b and Fos. The results are plotted as relative fold changes (RFC) with WT air (WTAir) as the baseline for three replicates (*n* = 3).

Thus, microarray data generated from CS-exposed mouse lungs can elucidate the regulation of gene expression in response to oxidative stress. In Figure 4, microarray data for a cross-section of three stated Nrf2 targets are summarized. Nqo1 and Sod1 have previously been identified as transcription regulatory targets of Nrf2 [3]. Als2 is a novel target arising out of the computational analysis being reported here. We performed a set of measurements showing upregulation of all three genes only in the presence of the Nrf2 gene (wild type; no knockout) and CS-induced oxidative stress (Figure 4). This is additional evidence for a regulatory role for Nrf2 in the expression of Nqo1, Sod1 and Als2. Furthermore, quantitative RT-PCR experiments were conducted on a gene set found to be differentially expressed in these CS exposure microarrays as well as identified by ARACNE or CLR as Nrf2 targets. The results affirm the regulatory role of Nrf2 for many of the gene targets predicted by our combined analysis of microarray and sequence data (Figure 5). Nqo1, Sod1, Ercc6, Prdx6, Als2, Txnrd2, Park7, Srxn1, and Epas1 all undergo enhanced upregulation after CS exposure only in the presence of the Nrf2 gene. Thus, we have good evidence from quantitative RT-PCR that Nrf2 positively regulates the expression of these genes. In the case of Sod2, Ppp1r15b and Fos, CS-induced upregulation is modestly enhanced in the absence of Nrf2. It is inferred that Nrf2 exerts a negative regulatory influence on the expression of these genes.

## Discussion

Rangasamy et al. list 45 genes whose expression increase in Nrf2^+/+^ mice but not Nrf2^−/−^ mice in response to CS exposure [16]. All but four of these Nrf2-dependent genes have the consensus Anti-oxidant Response Element (ARE) within 10 kilobases upstream of the transcription start site. Thirteen of the 45 are antioxidant, 14 are detoxifying enzymes, seven are protective proteins, two are transcription factors (TFs), three are transporters, two are phosphatases and one is a receptor [16]. The experiments did not distinguish between the direct regulatory gene targets of Nrf2 and those genes that are only indirectly dependent on Nrf2. Moreover the presence of the ARE is itself insufficient proof of Nrf2 regulation, the ARE being a composite site where several TFs interact [17].

The involvement of the ARE and Nrf2 in the regulation of the expression of genes involved in the response to oxidative stress has been noted [3]. We identified a total of nine genes as potential Nrf2 regulatory targets by all three computational methods—ARACNE, CLR, and LibSVM. Two of the nine (Nqo1 and Prdx1) have been previously found as Nrf2 targets, as reported above and in Table 2. This leaves a list of seven novel targets for Nrf2 regulation, reported across all three computational algorithms: Als2, Atf1, Nfkbib, Ppp1r5b, Prnp, Sod2, and Srxn1 Both manual and automated literature searches yielded no previous reports of these genes being direct targets of Nrf2 regulation. (Although Idh1 could be numbered among the targets based on the output of the ARACNE and LibSVM runs, it is not listed in Table 4 because the CLR runs did not establish a dependency between Idh1 and Nrf2 at the cut-off used for the other gene targets.) Also, while Park7 was identified only by ARACNE, and not by CLR and LibSVM, its gene expression measurements showed it as positively regulated by Nfr2 in our separate quantitative RT-PCR experiments, and hence is included in our discussion below.

### Genes Associated with Neurodegenerative Disorders

Several of the genes that were identified in our work as potential targets of Nrf2 transcriptional regulation in the mouse lung have been implicated in certain neurodegenerative disorders. Of the set of 46 genes of interest (see Methods), six are annotated in the Kyoto Encyclopedia of Genes and Genomes (KEGG) [18] with the “Neurodegenerative Disorders” classification. (Table S2 lists Gene Ontology (GO) [19] and KEGG annotation on these 46 genes.) These six are Crebbp, Apoe, Als2, Sod1, Park7, and Prnp. Five of these six, all except Apoe, were placed by our analysis in the list of 21 potential targets of direct Nrf2 regulation shown in Table 1. In addition, Nfe2l2 is annotated in KEGG as associated with prion disease. Using the LibSVM algorithm, we found that Nfe2l2 regulates itself (see Conclusions) and therefore is included in our set of 21 targets. Thus, of the seven genes marked in KEGG as associated with neurodegenerative disorders, six appear in our set of Nrf2 targets, roughly a two-fold enrichment of what we would expect to see by chance (7×21/46 = 3.2 genes).

Death of motor neurons induced by an Amyotrophic Lateral Sclerosis (ALS)-linked Sod1 mutant is prevented by the Als2 gene product, alsin [20]. Alsin acts as a guanine nucleotide exchange factor for Rac1 and Rab5, both GTPases [21],[22]. A number of protein function-altering Als2 mutations have been identified as causing ALS [23].

Human diseases associated with S-glutathionylation, a common post-translation modification, include PD, diabetes, hyperlipidemia, Friedreich's ataxia, renal cell carcinoma and HIV/AIDS [24],[25]. The oxidoreductase, Srxn1, plays a role in signaling by catalyzing reduction following S-glutathionylation [26]. Srxn1 is involved in reversing NO-induced protein glutathionylation; Srxn1 protein deglutathionylation results in the restoration of phosphatase activity of non-receptor-type protein tyrosine phosphatase [25]. Srxn1 also catalyzes the reduction of cysteine sulfinic acids [27].

Park7, also known as DJ-1, has been linked to a number of Parkinson's Disease (PD) pathways [28]. When oxidized, Park7 acts as a chaperone protein that prevents the characteristic aggregation of certain proteins in PD [29]. Indeed, oxidized forms of this protein accumulate in the brains of some PD and Alzheimer's disease patients [30]. Its functional integrity is so important that up to 1% of PD cases are associated with Park7 mutations [31].

The four traditional classes of prion diseases (Creutzfeldt–Jakob disease, kuru, fatal familial insomnia, and Gerstmann–Straussler–Scheinker syndrome) all involve mutations of Prnp and multiple abnormal conformations of its protein product Prp [32]. This set of neurodegenerative diseases has become intensely epidemiologically interesting following the transmission of bovine spongiform encephalopathy to humans and the apparent concomitant emergence of the variant Creutzfeldt–Jakob disease [33]. The ARACNE, CLR, and LibSVM runs in these studies all indicate a regulatory role of Nrf2 on the expression of the Prnp gene in the mouse lung (Tables 1 and 2).

Using the two algorithms (ARACNE and CLR), we establish direct statistical dependencies between the expressions of genes such as Sod1, Als2, Srxn1, and Park7, and the expression of Nfe2l2 (the Nrf2 gene) in the mouse lung. The LibSVM studies affirm that in the case of Als2 and Srxn1, the direct statistical dependencies indicate transcriptional regulation by Nrf2. Furthermore, our quantitative RT-PCR experiments show that CS-induced oxidative stress of the mouse lung increases the mRNA expression of several of these genes, and that these increases require the presence of Nrf2. Experimental evidence (Figures 4 and 5) confirms, for instance, that Als2 is indeed a novel Nrf2 target. Cigarette smoke (CS) exposure induces oxidative stress (Figure 3A and 3B) and acts as an inducer of Nrf2-mediated transcription. In wild type mice (but not Nrf2 knockout mice), increases in Sod1, Nqo1, and Als2 mRNA expressions are observed after CS exposure. These data point to a transcriptional regulatory role for Nrf2 on these genes in the mouse lung.

**Figure 6:**
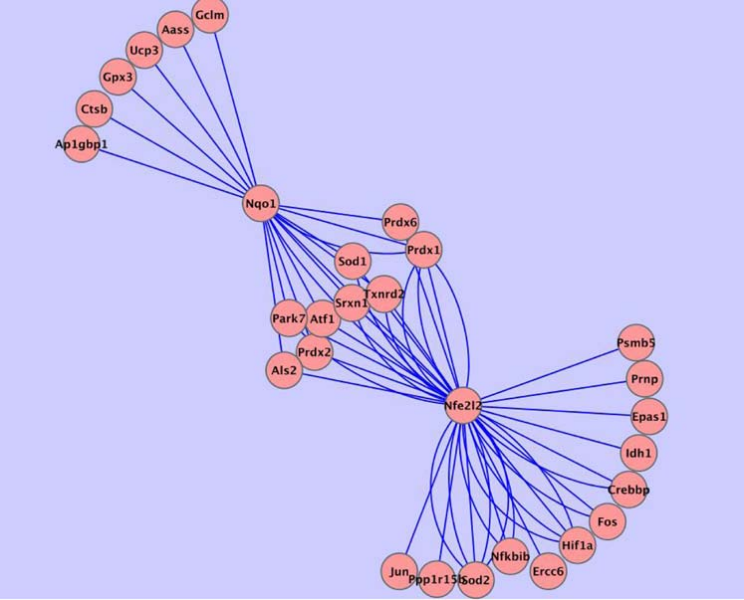
ARACNE algorithm results showing connections to genes Nqo1 and Nfe212 (Nrf2). Results of ARACNE runs on microarray data showing extension of the Nfe212 (Nrf2) network from Nqo1, one of its targets. Transcriptional regulatory interactions involving Nrf2 and Nqo1 as determined using the ARACNE algorithm. Across 260 microarrays, profiles of genes categorized by the Gene Ontology as participating in the *response to oxidative stress* were examined. The DPI tolerance was set at 0.15; *p* = 1e-7. The nodes represent genes and the lines (edges) between them represent transcription regulation relationships. Interactions involving Nrf2 (Gene Symbol: Nfe2l2) and Nqo1 (one of its regulatory targets) are depicted. Multiple edges between two nodes indicate multiple array probe-sets on the arrays referencing the same gene.

**Figure 7:**
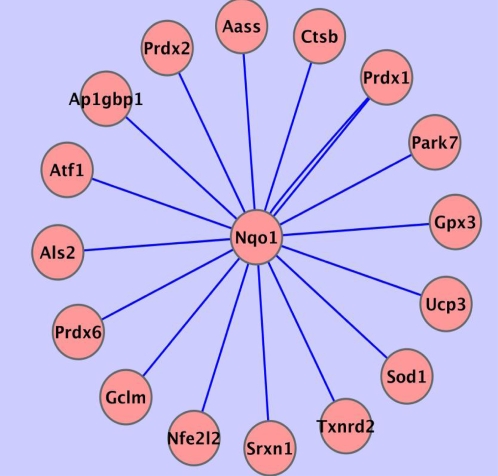
Alternate view of the ARACNE algorithm results, here focused on the subset of connections that directly involve Nqo1.

Nqo1, also known as DT-diaphorase or NAD(P)H:quinone oxidoreductase, was found to be a target of direct regulation by Nrf2 under both the CLR algorithm runs (Figure 1) and the ARACNE runs (Figure 2). In addition, the LibSVM prediction that Nqo1 is trans- criptionally Nrf2-regulated has biochemical proof [34]. Thus, Nqo1 was predicted to be a direct target of Nrf2 by all three methods, and has been confirmed in the literature as such. We therefore considered it suitable for expanding our study of the oxidative stress response beyond Nrf2. All direct dependencies (all edges) involving Nqo1 as determined by the ARACNE runs are shown in Figures 6 and 7. Although the current literature does not capture all the relationships being identified here, the edges represent a number of plausible regulatory or functional relationships, involving Nqo1. For instance, there is no previous finding of the direct relationship between Gpx3 (glutathione peroxidase 3) and Nqo1. However, Gpx3 is distributed in the same fashion as Nqo1 and Sod1 (Cu/Zn superoxide dismutase) [35],[36]. The relationship between Nqo1 and certain other connected nodes, such as Sod1, have been identified [37].

### The Algorithms Used and the Biological Significance of Mutual Information

Both CLR and ARACNE use the concept of mutual information (MI). Why not use Euclidean distance or Pearson correlation for pair-wise calculations, as is done in standard microarray-based gene clustering? Why use MI? Unlike Euclidean distance and Pearson correlation, MI does not assume that the relationship between the genes is linear. A major advantage of this information theoretic calculation is its nonparametric nature, and the entropy calculations performed in calculating the MI value do not require any assumptions about the distribution of variables. MI provides a general measurement for dependencies in the data: negative as well as positive, nonlinear as well as linear [38],[39].

The higher the MI score between two genes, the greater the information we derive on the states of the first gene from the pattern of states in the other, and the greater the likelihood that one of the genes is directly regulating the other. While both ARACNE and CLR are mutual information based algorithms, and while both were applied here to the same microarray datasets, we believe that there is a legitimate reason to conclude that a regulatory connection found by both algorithms is of higher probability of being a true regulatory relationship than if only one of the two algorithms scored such a connection highly. ARACNE and CLR impose a superstructure on the basic MI calculation that differs in important ways. ARACNE also post-processes the results in a different manner. Also, the binning (discretization) methods used are different—which can be highly important. Therefore when a gene-to-gene relationship is scored highly by both algorithms, the algorithms have arrived at that conclusion using different calculations. An analogy can be made here to the Oak Ridge National Laboratory GRAIL gene finder tool which uses several algorithms—operating on the same sequence data—and combines their results for improved gene calling. For our resource-limited, time-limited exploratory analysis, we focused on the inferred regulatory connections we believed had the highest probability of proving to be biologically valid and the most robust, that is, on the connections inferred by ARACNE and CLR together.

As with the standard clustering metrics, MI calculations are symmetric, yielding identical scores from gene A to gene B and from gene B to gene A. Therefore the directionality (which of the two genes regulates the other) cannot be inferred from the MI score alone. More information is needed: is one gene known or suspected to be a transcription factor? Does one of the two appear to connect (regulate, as a “hub”) many other putative targets? Does one gene connect (have a high MI score) to two or more putative target genes in the same operon? Additional information must be sought, with the regulatory edge in question looked at in the wider context of the entire inferred network.

Each edge connecting the nodes in Figures 1 and 2 is subject to one of at least two interpretations. First, we can interpret the edge as a direct dependency between the expression of a transcription factor producing gene and a target non-transcription factor gene, that is, as an indicator of direct transcriptional regulation of the target by the transcription factor via DNA binding of the transcription factor. For instance, in Figure 2 the edge between Nrf2 (gene Nfe2l2) and Sod1 depicts the fact that the expression of Cu/Zn superoxide dismutase (Sod1) is transcriptionally regulated by Nrf2 [40]. Second, if such an edge is one of two or more connections going into a common target, the source gene for one of those edges may be producing a protein necessary for the action of the primary transcription factor also connecting to the common target. For example, this would hold true for Nfe2l2 (Nrf2) and Park7 (Parkinson disease (autosomal recessive, early onset) genes as joint regulators of a common target gene. Park7 has no direct effect on Nfe2l2 mRNA levels. However, it does stabilize the Nrf2 protein produced by Nfe2l2, and is required for the transcriptional activity of Nrf2 [41], and thus, through binding to Nrf2 (rather than directly to DNA near the target), Park7 also regulates each of Nrf2's direct targets, with such regulation being reflected in the correlation between Park7 expression levels and that of the target of Nrf2.

In Figure 2, the edge between Nfe2l2 and Park 7 shows that Nrf2 also exerts regulatory control on the mRNA expression of Park7 itself. Microarray and quantitative RT-PCR data generated from Nrf2 knockout mice (Figures 4 and 5) show CS-induced enhanced mRNA expression of Nqo1, Sod1, Ercc6, Prdx6, Als2, Txnrd2, Park7, Srxn1, and Epas1 in wild-type but not Nrf2-knockout mice. In the knockout mice, where Nrf2 is absent, mRNA expression for these genes is dramatically decreased in response to CS (state of Nrf2 knockout CS-exposed “NOCS” in Figures 4 and 5) as compared to wild type with Nrf2 present and active. Thus we infer that Nrf2 is required for the CS-induced increase in Park7 mRNA expression. This assertion holds also for Nqo1, Sod1, Ercc6, Prdx6, Als2, Txnrd2, Srxn1, and Epas1, as can be seen in the figures.

As noted above, some of the genes reported (Park 7, Jun, and Crebbp) have been investigated and have been found to work with Nrf2, though they have not previously been identified as genes directly activated by Nrf2. They remain possible targets of Nrf2 regulation, with a possible fit into the category of feed-forward loops discussed below. Indeed we show that in the absence of Nrf2, CS elicits a suppression of Park7 and Jun mRNA expression (see state “NOCS” in Figure 5). Thus the significant mutual information content reported by ARACNE and CLR between each of these genes and Nfe2l2 has biological significance.

Edges between the genes producing two transcription factors are subject to similar interpretations as outlined above. In the first case, one of the two transcription factors is a transcriptional regulator of the gene producing the other. In the second case, such an edge can be an indication that the two transcription factors act as coregulators of the expression of other genes, with both proteins working closely together for properly modulated expression of the gene target(s), causing a very tight correlation in their gene expression patterns. These are not mutually exclusive categories. For example, Nrf2 and the transcription factor Atf1 can jointly regulate the target gene ferritin H, *and*, as our data indicate, Nrf2 can also be a transcriptional activator of Atf1. In fact, this is common triangular regulatory motif, called a *feed-forward loop* [42], between three genes in a transcriptional regulatory network.

### Feed-Forward Loops

Such connected subsets of three genes can often form what are known as feed-forward loop (FFL) transcriptional regulatory network motifs. These FFL motifs appear in hundreds of gene systems. In this context, gene Nfe212 (Nrf2) would be one of the three genes in an FFL subgraph, having an edge to Nqo1 as an activating regulator of that gene. The direction of the edges from Nqo1 to X, and from X to Nfe2l2 remain to be determined, as well as type of regulation for those two edges–activation or repression.

Other examples of possible feed-forward loops are as follows: (1) The gene product Jun (whose gene is shown in Figure 2 as connected to the Nrf2-producing Nfe212 gene by a high ARACNE score), is part of the activator protein 1 (AP1) transcription factor and is known to serve as a coregulator with Nrf2 in some promoter regions [6]. (2) The Fos gene product was found to be connected to Nrf2 by high scores using both the CLR algorithm (Figure 1) and the ARACNE algorithm (Figure 2). Fos can be a component of AP1 and has been shown to negatively regulate ARE-mediated transcription regulation [43]. (3) Another example is activating transcription factor 1 (Atf1), shown connected to Nrf2 in both Figures 1 and 2. Also, there is a recent report by Iwasaki et al. [44] indicating Atf1 is a transcriptional repressor at an anti-oxidant response element, thus modulating target response to Nrf2, which is the principal transcriptional activator of the antioxidant response element.

**Figure 8:**
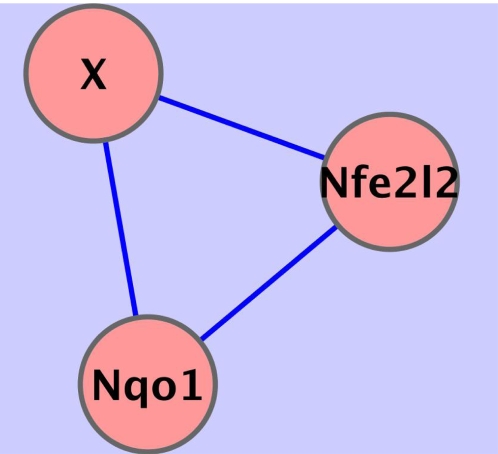
A subset view of three-party direct dependencies involving Nrf2 (Nfe2l2), Nqo1 and gene “X” from the ARACNE algorithm results. Gene X is a placeholder for any of these genes: Sod1, Srxn1, Txnrd2, Prdx1, Prdx2, Prdx6, Atf1, Park7 and Als2. Nrf2 transcriptionally regulates Nqo1 expression; this defines one of the three edges.

The regulatory network shown in Figure 6 has inherent within it a number of three-party relations of the kind characterized in Figure 8, where the edge between Nrf2 and Nqo1 represents transcriptional regulation by Nrf2. However the edge between Nrf2 and gene X in Figure 8 (with X representing any of the following: Sod1, Srxn1, Txnrd2, Prdx1, Prdx2, Prdx6, Atf1, Park7, or Als2) and the edge between X and Nqo1 represent a number of possible transcriptional regulatory relationships, with one gene serving as a final target, and the other two genes functioning as activators or repressors of mRNA expression.

**Figure 9:**
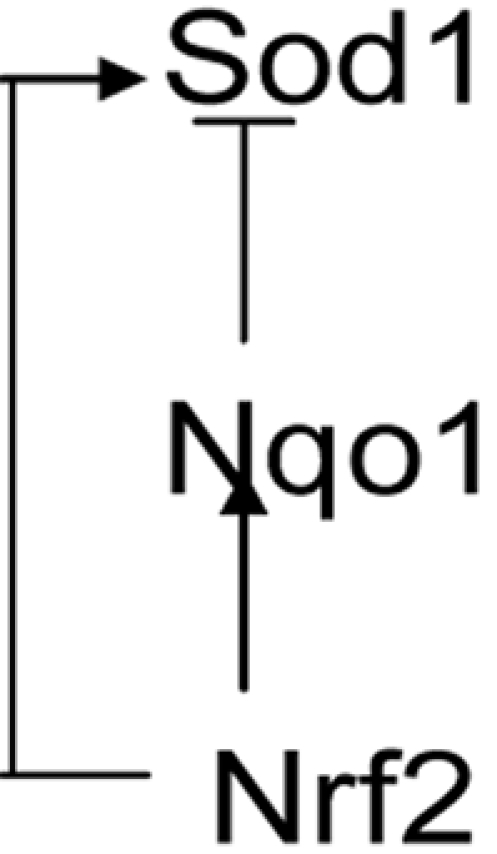
ARACNE algorithm results and a possible feed-forward loop. A depiction of a possible feed-forward loop involving Nrf2, Sod1 and Nqo1 captured in the networks generated using ARACNE on microarray data. In order to assign directionality to the edges of the generated subgraph, there is a need for biological context: Nrf2 transcriptionally regulates both Sod1 and Nqo1. In lung epithelial cells (A549-S), inhibition of Nqo1 gives the same effect on the generation of hydrogen peroxide by low dose quinones as the introduction of exogenous Sod1 [37]. Inference: Nqo1 is a repressor of Sod1.

Uri Alon [45] has classified the possible feed-forward loops within such a three-node, three-edge relationship into eight types. In the specific case of Nrf2-Nqo1-Sod1, the transcriptional regulatory influence of Nrf2 on both Nqo1 and Sod1 has been established. Hence we have activation edges from gene Nfe212 (Nrf2) to both Nqo1 and Sod1. The remaining, less characterized edge represents the Nqo1–Sod1 regulatory relationship. Does Nqo1 directly regulate Sod1—or vice versa? Watanabe et al. [37] report that inhibition of Nqo1 in lung epithelial (A549-S) cells results in inhibition of H_2_O_2_ generation by quinones. Exogenous Sod1 also inhibits H_2_O_2_ generation by low levels of quinones. Thus inhibition of Nqo1 has the same effect as raising the level of Sod1. Based on this, we infer that Nqo1 exerts an inhibitory effect on Sod1. Hence, if Nqo1 is increased, Sod1 should be repressed, and H_2_O_2_ generation will not be inhibited. And, therefore, if Nqo1 is inhibited, then H_2_O_2_ generation will be inhibited, agreeing with experimental observation. This inferred relationship, resolving the character of the remaining edge, is illustrated in Figure 9. The subgraph shows an connection from Nqo1 to Sod1, with Nqo1 acting as an inhibitor. This matches the type 1 incoherent feed-forward loop, which is one of the two most frequent occurring of the eight types of FFLs. (The other common type is the type 1 coherent feed-forward loop, where all three edges represent transcriptional activation.)

**Figure 10:**
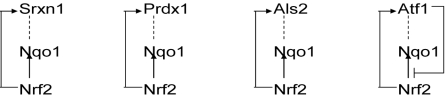
Predicted feedforward loops involving Nrf2, Nqo1, Srxn1, Prdx1, Atf1, and Als2. On the basis of LibSVM predictions (Tables 1 and 3), Nrf2, Nqo1, Srxn1, Prdx1, Atf1 and Als2 are all regulatory targets of Nrf2 transcriptional activity. ARACNE runs on 260 microarray data indicate direct dependencies between Nqo1 and Srxn1, Prdx1, Atf1, Als2 and Nrf2. In addition, there is evidence Atf1 acts as a transcriptional repressor on the anti-oxidant response element of another promoter [44]. This figure captures these relationships. Transcriptional regulatory relationships are depicted by arrows and less well defined relationships are depicted with hidden detail (dotted lines).

Four possible Nrf2-Nqo1-X feed-forward loops are shown in Figure 10. The third gene, gene “X”, can be any one of Srxn1, Prdx1, Atf1, or Als2. All four putative loops have two defined edges, both of which represent transcriptional activation by Nrf2. However, the third edge, corresponding to a direct regulatory relationship between Nqo1 and gene X, remains to be established. All of these genes are involved in the response to oxidative stress, however. For example, Als2 knockout mice are more susceptible to oxidative stress, and Als2 protects against oxidative stress [46],[47]. On the basis of the results of our computational analysis, we believe that additional work to confirm direct regulatory relationships between Nqo1 and Srxn1, Prdx1, Atf1, or Als2 would be warranted.

### Noise

As explained in the Data Sources sub-section under Methods, data samples, all from mouse lung, were run on two platforms: the Affymetrix GeneChip Mouse Genome 430 2.0 array and the Affymetrix Mouse Expression Set 430 (MOE430A). The latter is a subset of the former. However, for each of our network inference runs data from only a single platform was used, not both. While this limited the number of data points on each gene to something less than if we had combined the two platforms, we thus avoided the problem of comparing gene expression across platforms. The remaining task was that of combining data from multiple laboratories that employ the same microarray platform. (Table S1 lists the data sources.) We performed RMA analysis using the affy package in BioConductor, as stated in Data Sources section of Methods. We acknowledge that noise will be introduced when combining array sets from different sources and that this could be a confounding factor. However, we stress that we were functioning in the framework of relatively low-cost, relatively simple exploratory analysis, mining the growing collection of public microarray datasets for identification of candidate regulatory relationships to be later confirmed via LibSVM, quantitative RT-PCR, and literature search. And, hopefully, we are serving as an example of what can be done, with relatively modest cost, in analysis of such datasets, with our work having general application for other researchers analyzing transcriptional regulatory networks. Our working assumption was that multiple-source introduced noise/bias, while hiding regulatory edges whose correlations in gene expression could not rise above such noise, would not prevent at least some true regulatory connections—the strongest ones—from being found by the CLR and ARACNE algorithms. We believe that our assumption bore fruit.

### Conclusions

In the set of 21 genes reported out by one or more of our three algorithms (CLR, ARACNE, and LibSVM) with empirically determined high confidence thresholds, shown in Table 1, four have been verified in the literature as Nrf2 activation targets: Nqo1, Prdx1, Sod1, and Nfe2l2 itself (postive autoregulation). Two of these four, Nqo1 and Prdx1, were reported by all three algorithms. Sod1 was reported out by ARACNE, and Nfe2l2 (Nrf2) was reported out by LibSVM (such autoregulation being undetectable by the other methods). Ten more possible gene targets were found by one or more of the algorithms where the literature shows that the product of the gene interacts with Nrf2 as coactivators or coregulators (Table 2). These ten genes remain as possible Nrf2 targets in the context of the formation of feed-forward loops. Lastly, seven of the 21 were reported out by all three algorithms, but with no literature evidence linking them to Nrf2 (Table 4). Hence this is the first report of these seven genes (Als2, Atf1, Nfkbib, Ppp1r15b, Prnp, Sod2, and Srxn1) as being strong candidates for direct targets of Nrf2 activation in the mouse lung.

Separate RT-PCR experiments indicate that Nrf2 positively regulates the expression of the Nqo1, Sod1, Ercc6, Prdx6, Als2, Txnrd2, Park7, Srxn1, and Epas1 genes in the mouse lung (Figure 5). In addition, these experiments indicate that Nrf2 may negatively regulate the expression of the Sod2, Ppp1r15b, and Fos genes. Thus these pieces of experimental evidence affirm several inferences made using the CLR, ARACNE, and LibSVM algorithms.

We believe our work shows the usefulness of network inference algorithms such as CLR and ARACNE on the growing body of microarray data. Using such algorithms and datasets, exploratory analysis is now possible that can usefully guide laboratory work with a relatively modest effort.

Finally, in addition to identifying putative targets of Nrf2, we extended our analysis of the network downstream of Nrf2 by identifying probable feed-forward loops involving Nqo1, one of the Nrf2 regulatory targets. We believe further extension of our analysis downstream of Nrf2 is possible, and hope to continue work in this area.

## Methods

### Quantitative RT-PCR

Total RNA was extracted using RNAeasy kit from Qiagen according to the manufacturer's instructions, and 2 µg of total RNA was used for cDNA synthesis. Quantitative PCR analyses were performed by using assay on demand probe sets commercially available from Applied Biosystems. Assays were performed by using the ABI 7000 Taqman system (Applied Biosystems). GAPDH was used for normalization. The cycle threshold (*C*_T_) value indicates the number of PCR cycles that are necessary for the detection of a fluorescence signal exceeding a fixed threshold. The fold change (FC) was calculated by using the following formulas: Δ*C*_T_ = *C*_T_(GAPDH)−*C*_T_(target gene) and 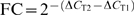, in which Δ*C*_T1_ represents the highest *C*_T_ value among all the samples and ΔC_T2_ represents the value of a particular sample. Results are expressed as mean values of relative fold changes (RFC) for *n* = 3 with WT Air as the baseline.

### Total Glutathione Assay

Total glutathione was determined using a modified Tietze method by measuring reduction of 5,5′-dithiobis-2-nitrobenzoic acid in a GSR-couple assay [48].

### TBARS Assay

Thiobarbituric acid reactive substances (TBARS) as a measure lipid peroxidation was assessed by the method of Ohkawa et al. [15].

### Information-Theoric Network Inference Algorithms

#### CLR

The first algorithm we employed is the Context Likelihood of Relatedness (CLR) algorithm [8] from the Gardner group at Boston University. CLR uses a novel method for estimating the likelihood of the MI score between two genes that is dependent upon the selected gene pair (and, hence, yields a “context likelihood” value). As with our second algorithm, described below, CLR starts by calculating a matrix of MI values between all the Affymetrix probe sets. However, it then estimates the likelihood of the MI score between genes A and B by comparing the MI score to a background distribution of MI values. This background distribution is created anew for each pair of genes from their two sets of MI values against all other genes in the set. The Gardner group believes that the sparseness of biological regulatory networks, with most MI scores representing random background from indirect network relationships, allows us to approximate the MI scores as independent variables, and thus use a joint normal distribution as an estimate of the true background distribution for the combined set of MI values for genes A and B. If *Az* is the *z*-score of the MI score between gene A and gene B in gene A's MI score distribution, and *Bz* is the *z*-score of the MI score between gene A and gene B in gene B's MI score distribution, then the CLR value (likelihood estimate) produced between genes A and B is set to:
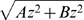
Thus, the CLR score between any pair of genes is set in a local context, where the background distribution arises from the mutual information of all the possible incoming and outgoing edges for each gene of the pair. One other item of note: each of two MI algorithms we employed requires discretization (binning) of the gene expression values. CLR uses B-spline functions for such binning, a recent innovation in this context reported in Daub et al. [49].

#### ARACNE

The second algorithm used is the Algorithm for the Reconstruction of Accurate Cellular Networks (ARACNE), and comes from the Califano group at Columbia University [9]–[11]. The two most important customizable parameters for the MI calculation in ARACNE are (1) the kernel width of the Gaussian estimator (used in ARACNE's “accurate” mode, as compared to its “fast” mode, which uses a simpler binning method), and (2) the MI threshold or p-value that is used to assess whether a MI value is statistically significant enough for its score and associated gene pair to be reported in the output. A default value is calculated by ARACNE for the kernel width parameter, depending upon the size and statistics of the dataset. ARACNE follows its set of MI calculations with an optional postprocessing step that is used to eliminate interactions that are likely to be indirect. This additional processing step uses an information-theoretic property to remove indirect regulatory influences that are incorrectly appearing due to having high enough MI scores to be recorded as direct edges, that is, as directly interacting genes. This information-theoretic property is called the data processing inequality (DPI) [12]. Calculations for the DPI require an accurate estimation of MI ranks, which in turns requires an additional ARACNE parameter called the “DPI tolerance”. The DPI is used by ARACNE to compensate for errors in the estimate that might affect these ranks. ARACNE's developers have found that a tolerance of between 0% and 20% (0 to 0.20) yields the best results; higher values tend to cause high false-positive rates. (Setting the tolerance to 100% would mean that the DPI post-processing is not used, and all regulatory edges found would be accepted.)

### Support Vector Machine Classification Algorithm

Support Vector Machines (SVMs) are a set of supervised machine learning techniques that lie in the family of generalized linear classifiers. They employ a training set, with the SVM classification results scored against the known data classification values, and with the SVM parameters iteratively refined against that metric [50]. SVMs are trained to separate the given binary labeled training data with a hyperplane that is maximally distant from them. After training, the SVM is used to classify new data. SVMs are relatively new, but have already been used extensively in bioinformatics due to their robust performance in classification on sparse and noisy datasets. For our analysis, we used our (trained) SVM to identify genes belonging to the set of gene targets directly regulated by Nrf2. The binary labeled training data was the set of upstream promoter regions from a set of gene targets known to be directly regulated by Nrf2, combined with the set of promoter regions from a set of genes known not to be directly controlled by Nrf2. The binary classification to be learned was: target/not target. Thus, the object was to train the SVM to detect those genes that are candidates for targets of direct regulation by Nrf2, based on the classification the SVM makes from its analysis of the base composition in the upstream promoter region of the candidate. The SVM implementation we used is the LibSVM from Chang and Chih-Jen [13].

### Data Sources

Publicly available mouse lung microarray data from seven disparate laboratories were employed, as well as data from the Biswal lab. In all, 260 Affymetrix CEL files from two platforms, Affymetrix GeneChip Mouse Genome 430 2.0 array and the Affymetrix Mouse Expression Set 430 (MOE430A), were collected. Of these, 224 arrays were obtained from the publicly available Gene Expression Omnibus Datasets (Table S1). These mouse lung arrays represent a variety of perturbations of the lung protein-protein interaction network, including gene knockout and ligand treatment. From an R command line (http://cran.r-project.org/), the affy package of BioConductor (http://www.bioconductor.org/) was used to perform Robust Multi-array Average (RMA) analyses on the datasets [51]. The process consisted of the microarray data being normalized and log-transformed, following background correction, according to the method of Irizarry et al. [51]. The RMA analyses were performed on four subsets of the array samples gathered:

- the 71 publicly available GeneChip Mouse Genome 430 2.0 arrays (Table S1, in Microsoft Excel spreadsheet format)
- the 36 Biswal Lab GeneChip Mouse Genome 430 2.0 arrays [52]
- a pooled dataset consisting of the 36 Biswal Lab arrays and the 71 public arrays
- the 153 publicly available MOE430A arrays (Table S1)

From the tables generated, data for probe sets representing genes classified under “response to oxidative stress” from the Gene Ontology [19] were then selected. The contents of this class of thirty six genes identified under the GO identifier GO:0006979 are: Aass, Als2, Apoe, Cat, Cln8, Ctsb, Cygb, Epas1, Ercc2, Ercc6, Gab1, Gclm, Gpx1, Gpx3, Hif1a, Idh1, Mtf1, Nme5, Nqo1, Nudt15, Oxsr1, Park7, Ppp1r15b, Prdx1, Prdx2, Prdx6, Prnp, Psmb5, Sod1, Sod2, Srxn1, Tcf1, Txnip, Txnrd2, Xpa, and Ucp3. In addition, the following relevant possible transcription regulators were added: Nfe2l2, Ap1gbp1, Atf1, Creb1, Crebbp, Fos, Hsf1, Jun, Rela, and Nfkbib. The selection was facilitated by a parser we wrote in Lisp [53] for this purpose.

All further analyses were confined to these oxidative stress response gene subsets, using four different methods to find direct regulatory targets of Nrf2. The CLR and ARACNE algorithms were used to examine gene expression patterns in the subsets, and to establish direct dependencies between the expressions of the specified genes and transcription factors such as Nrf2. The LibSVM utility in the Weka workbench [54],[55] was used to independently identify, using separate sequence-level data, transcriptional regulatory targets of Nrf2 among the putative Nrf2 target genes returned by the CLR and ARACNE algorithms. This identification was based on a comparison of the promoter regions of the genes to those of known Nrf2 targets. For our fourth analysis method, we matched results from the first three algorithms against Nrf2 gene targets in networks generated using automated literature searches by way of the Agilent Literature Search plug-in [56] of the Cytoscape network visualization platform [57].

### CLR Runs

We used an implementation of the CLR algorithm within the Software Environment for BIological Network Inference (SEBINI) workbench [58],[59]. The CLR binning parameters were set to use 10 bins, with a spline degree of 3. The CLR values were converted to *z*-scores within the SEBINI platform, and a *z*-score cut-off of 2.0 was then employed to select the highest scoring potential regulatory edges. The putative regulatory edges were outputted from SEBINI in Cytoscape Simple Interaction Format (SIF) and viewed and analyzed in Cytoscape and CABIN (as was done with the ARACNE output).

### ARACNE Runs

The *p*-value for establishing that the mutual information between gene pairs was significant enough to report out was set at 10^−7^. The percentage of MI estimates considered as sampling error (the DPI tolerance) was set at 0.15. A parser was written in Lisp to convert the outputs into the SIF file format. Each set of edges was thus represented as a network within Cytoscape and CABIN for further analysis. Interactions involving the transcription factor Nrf2 were selected out and entered into Cytoscape and CABIN as smaller-sized networks, for simpler visualization of our Nrf2-based analysis.

### LibSVM Runs

As detailed in Table S3, a set of 26 known Nrf2 targets [3] were used for the generation of the true positive part of the LibSVM training set. A set of 23 genes determined to be *not* Nrf2-regulated [60] formed the true negative part of the training set. The LibSVM Support Vector Machine implementation in the Weka workbench was used for these studies [54],[55] The results (Table 3) were obtained with normalized data on the nu-SVC classifier, the Radial Basis Function: exp(−γ*|*u*−*v*|^2^) kernel type, with *ν* = 0.36, *γ* = 2^−13^, cost = 1. and training set size = 49.

Details on the structure of the LibSVM datasets used are described in Text S1. Promoter sequences consisting of 1,000 nucleotides upstream to 100 nucleotides downstream for each gene were obtained from the Gene Sorter (http://www.genome.ucsc.edu). For each promoter sequence, a vector of size 308, with elements characterizing features of the sequence, was generated using Common Lisp code. The elements of the vector included a Boolean value indicating whether or not the Antioxidant Response Element (ARE) to which Nrf2 binds to activate gene transcription was present. The vector also included numbers characterizing the base pairs stretching between the ARE and the Transcription Start Site (TSS), the ARE and the TFIID bind site, the ARE and the Maf bind site, the ARE and the ATF4 bind site, the ARE and the cAMP Response Element (CRE), and the ARE and the TPA Response Element (TRE). For these characterizations, the three kinds of features used were *Composition*, *Transition* and *Distribution*. *Composition* is a reference to the proportions of nucleotide base types contributing to the promoter sequence make up. *Transitions* represent the frequency with which specific nucleotide base types are followed or preceded, within the sequence, by other nucleotide base types. *Distribution* is a statement concerning the dissemination of specific nucleotide base types within portions of the sequence (or the entire sequence). The data generated was formatted for use within the Weka Workbench software toolkit of machine learning packages in Java [54].

### Inference Based on Scientific Literature

We used the Agilent literature search tool to conduct literature searches [56]. This tool is available as a plug-in for the open source network visualization and analysis tool Cytoscape. It is used to create an inferred network based on published scientific literature for the proteins of interest. The Agilent Literature Search tool takes a protein list and searches for abstracts in several text engines. These search engines include those of the U.S. Patent Office and the National Center for Biotechnology Information (PubMed). The tool parses the search engine output to extract interactions and displays the resulting protein-protein interaction network as a graph within Cytoscape. Literature based evidence is a well recognized way of corroborating interactions detected by other computational prediction methods. Networks found via ARACNE, CLR, and LibSVM were compared to networks identified via this method in order to identify previously identified interactors with Nrf2 and separate out novel Nrf2 targets.

### Collective Analysis of Biological Interaction Networks (CABIN)

**Figure 11:**
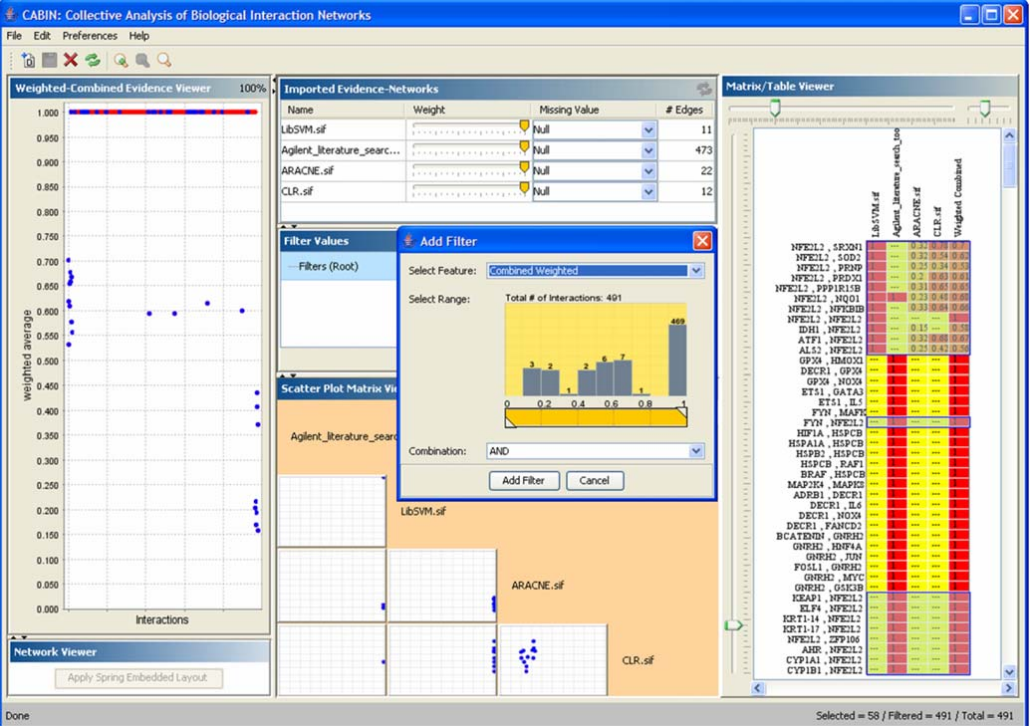
A depiction of analyses across networks. Use of the CABIN tool to conduct exploratory analysis for comparison and integration of interactions evidence obtained from the ARACNE and CLR algorithms along with the promoter region analysis using LibSVM and interaction evidence obtained using the Agilent Literature Search tools. The interactions involving Nrf2 are selected and highlighted in blue.

As indicated under “Data Sources” above, four sets of RMA-analyzed microarray data constituted the source of four networks for each of the algorithms used. These networks were inputs into the CABIN tool [14], which is also available as a plug-in for Cytoscape. CABIN was thus used to analyze, compare and merge the inferred networks obtained using ARACNE, CLR, LibSVM and Agilent literature search. CABIN provides the ability to assign weights or confidence to an inferred network, choosing cutoffs by applying dynamic filters. It also provides multiple viewers depicting different abstractions of the data. In this study, the multiple coordinated viewers within CABIN fostered comparison of inferred networks obtained using the algorithms ARACNE, CLR and LibSVM. These networks were further corroborated by combining literature based evidence obtained using the Agilent Literature search tool. Such combined network analysis within CABIN is demonstrated in the screen snapshot shown in Figure 11.

### Microarray Experiments

Microarray experiments were conducted with CD-1 Nrf2 wild type (WT) and Nrf2 knockout (NO) mice exposed to either five continuous hours of cigarette smoke (CS) or twenty four hours of air. For the purpose of such studies, approximately 5 hours of continuous CS exposure is about equivalent to one day of cigarette smoking [16]. In the CS-exposed group, there was immediate sacrifice and lung collection after cessation of smoke exposure. In the other group, age-matched air-exposed mice were killed with immediate lung collection following sacrifice. Total RNA was isolated using the Qiagen protocol (Qiagen Inc.). The cDNA was synthesized and Affymetrix microarray (Mouse genome 430A 2.0 array) was conducted as previously shown [16]. Scanned output files were analyzed by using Affymetrix GeneChip Operating Software version 1.3 and were independently normalized to an average intensity of 500. Further analyses were done as described previously [52]. In addition, the Mann-Whitney pairwise comparison test was performed to rank the results by concordance as an indication of the significance (*P*≤0.05) of each identified change in gene expression. The results for Sod1, Nqo1 and Als2 indicating mean (three replicates; *n* = 3) mRNA expression data from the microarrays are shown in Figure 4. (WT air exposed (WTAir), WT CS-exposed (WTCS), Nrf2 knockout air-exposed (NOAir) and Nrf2 knockout CS-exposed (NOCS)).

## Supporting Information

Text S1 Components of instance vectors used for machine learning.
(0.04 MB DOC)

Table S1 Listing of microarray data sources.
(0.03 MB XLS)

Table S2 Gene symbols, Entrez IDs, and functions.
(0.03 MB XLS)

Table S3 Genes used For machine learning.
(0.03 MB DOC)
