# Supplementary material for: Network Inference Algorithms Elucidate Nrf2 Regulation of Mouse Lung Oxidative Stress
Source: PLoS Comput Biol. 2008 Aug 29;4(8):e1000166. doi: 10.1371/journal.pcbi.1000166 (PMC2516606; doi:10.1371/journal.pcbi.1000166)
Supplement: Text S1 — Components of instance vectors used for machine learning. (0.04 MB DOC) [file pcbi.1000166.s001.doc]

**SUPPLEMENTAL TEXT 1**

**COMPONENTS OF INSTANCE VECTORS USED FOR MACHINE LEARNING**

Nucleotide Base Types: For the purpose of the sequence characterizations just described nucleotide bases were grouped based on whether they were purine or pyrimidine, the strength with which they form hydrogen bonds, and whether or not they were “keto” or “amino”:

The breakdown of the elements of each vector is as follows: percent Compositions for the individual nucleotide bases (positions 1 to 4); percent Compositions, Transitions, and Distributions for the Purine versus Pyrimidine base types (positions 5 – 17, consisting of two positions for Compositions, one for Transitions, and ten for Distributions); percent Compositions, Transitions, and Distributions for Strong versus Weak Hydrogen Bonding base types (positions 18 - 30, consisting of two positions for Compositions, one for Transitions, and ten for Distributions), percent Compositions, Transitions, and Distributions for “Keto” vrs “Amino” base types (positions 31 - 43, consisting of two positions for Compositions, one for Transitions, and ten for Distributions). The presence or absence of an ARE was indicated by a “1” or a “0” respectively at position 44. The sub-sequence made up of the stretch of bases between the ARE and the TSS was characterized at positions 45 through 88. At position 45, the “distance” was stated. In the absence of an ARE, the entire promoter sequence was characterized in *lieu* of the sought sub-sequence. In other words, in the absence of an ARE as defined above, the “distance” was longer. Details for positions 46 through 48 were as follows: individual nucleotide base percent Compositions were indicated at positions 46 -49; Purine versus Pyrimidine base type data were at positions 50 - 62; Strong versus Weak Hydrogen Bonding base type data were at positions 63 - 75; “Keto” versus “Amino” base type data were at positions 76 – 88. Correspondingly, the sub-sequence made up of the stretch of bases between the ARE and the TFIID bind site was similarly characterized at positions 89 through 132; that between the ARE and the Maf-bind site was characterized between positions 133 through 176; that between the ARE and the ATF4-bind site was characterized between positions 177 through 220; that between the ARE and the CRE was characterized between positions 221 through 264; that between the ARE and the TRE was characterized between positions 265 through 308.
