# Supplementary material for: Network Inference Algorithms Elucidate Nrf2 Regulation of Mouse Lung Oxidative Stress
Source: PLoS Comput Biol. 2008 Aug 29;4(8):e1000166. doi: 10.1371/journal.pcbi.1000166 (PMC2516606; doi:10.1371/journal.pcbi.1000166)
Supplement: Table S3 — Genes used For machine learning. (0.03 MB DOC) [file pcbi.1000166.s004.doc]

**SUPPLEMENTAL TABLE 3**
